# Supplementary material for: Neurophysiological Changes Induced by Music-Supported Therapy for Recovering Upper Extremity Function after Stroke: A Case Series
Source: Brain Sci. 2021 May 20;11(5):666. doi: 10.3390/brainsci11050666 (PMC8161385; doi:10.3390/brainsci11050666)
Supplement: Supplementary file 1 [file brainsci-11-00666-s001.zip › brainsci-1182511-supplementary.pdf]

Table S1. Demonstrates the performance on subsets of Jebsen hand function test.

Jebsen Hand Function Test subsets	P1						P2					
	**Base**	**Pre**	**Post**	**%**	**F-up**	**%**	**Base**	**Pre**	**Post**	**%**	**F-up**	**%**
**Subset 2**	8.3	7.1	6.3	-18%	5.7	-26%	14.4	15.0	12.9	-12%	11.8	-20%
**Subset 3**	16.2	16.3	12.0	-26%	10.2	-37%	23.2	19.9	18.5	-14%	19.9	-8%
**Subset 4**	13.7	9.3	10.4	-10%	11.6	1%	18.9	17.2	14.7	-19%	16.0	-11%
**Subset 5**	5.8	6.8	7.7	22%	5.4	-14%	17.6	16.2	15.8	-7%	13.9	-18%
**Subset 6**	5.6	5.2	5.3	-2%	5.1	-6%	13.2	11.3	8.4	-31%	7.2	-41%
**Subset 7**	5.4	5.9	5.1	-10%	4.7	-17%	8.7	8.0	5.9	-29%	6.6	-21%

Subset 2: turning over a 3×5-inch card, Subset 3: picking up small common objects, Subset 4: simulated feeding, Subset 5: stacking checkers, Subset 6: picking up large light cans, Subset 7: picking up large heavy cans, F-up: Follow up

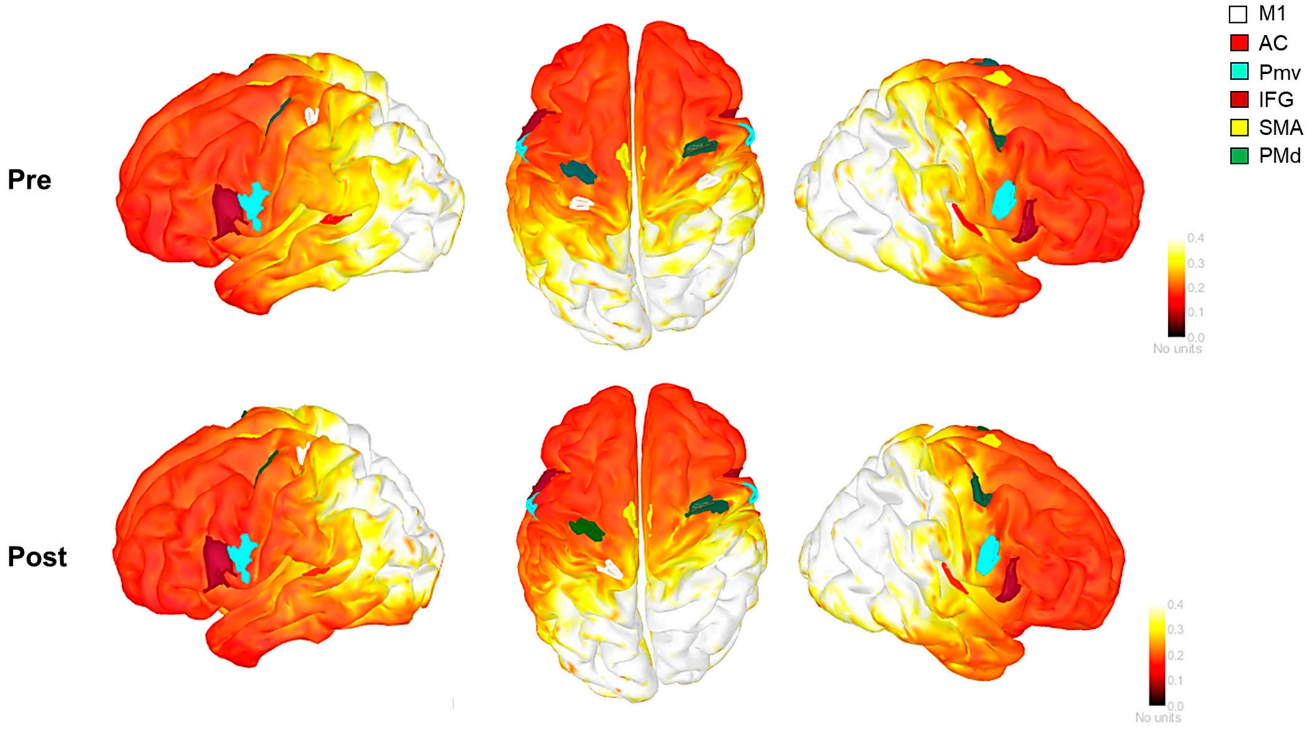

Figure S1. Relative power spectrum density maps for α-band in Participant 1. (M1: primary motor cortex, AC: auditory cortex, IFG: inferior frontal gyrus, SMA: supplementary motor area: PMv: premotor ventral cortex, PMd: premotor dorsal cortex, pre: before music support training, post: after music supported training, post-pre: difference in PSD for the post MST phase and the pre MST phase)

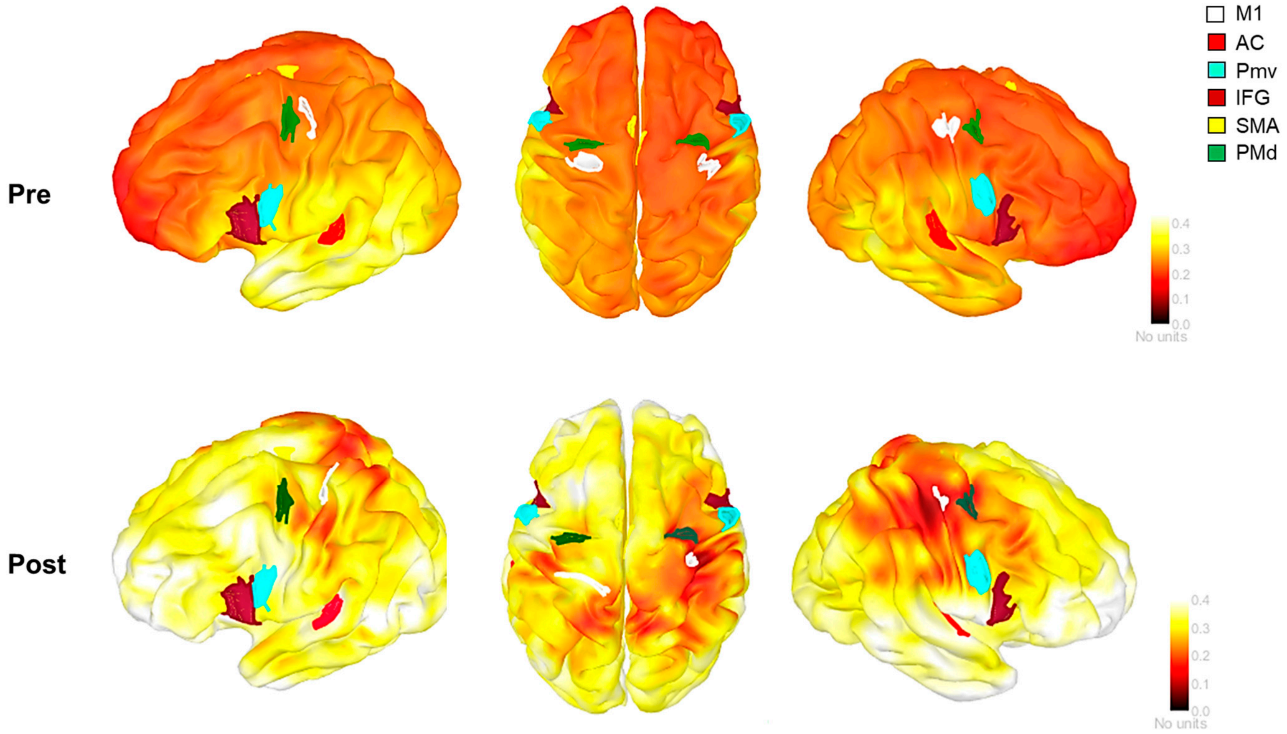

Figure S2. Relative power spectrum density maps for α-band in Participant 2. (M1: primary motor cortex, AC: auditory cortex, IFG: inferior frontal gyrus, SMA: supplementary motor area: PMv: premotor ventral cortex, PMd: premotor dorsal cortex, pre: before music support training, post: after music supported training, post-pre: difference in PSD for the post MST phase and the pre MST phase).

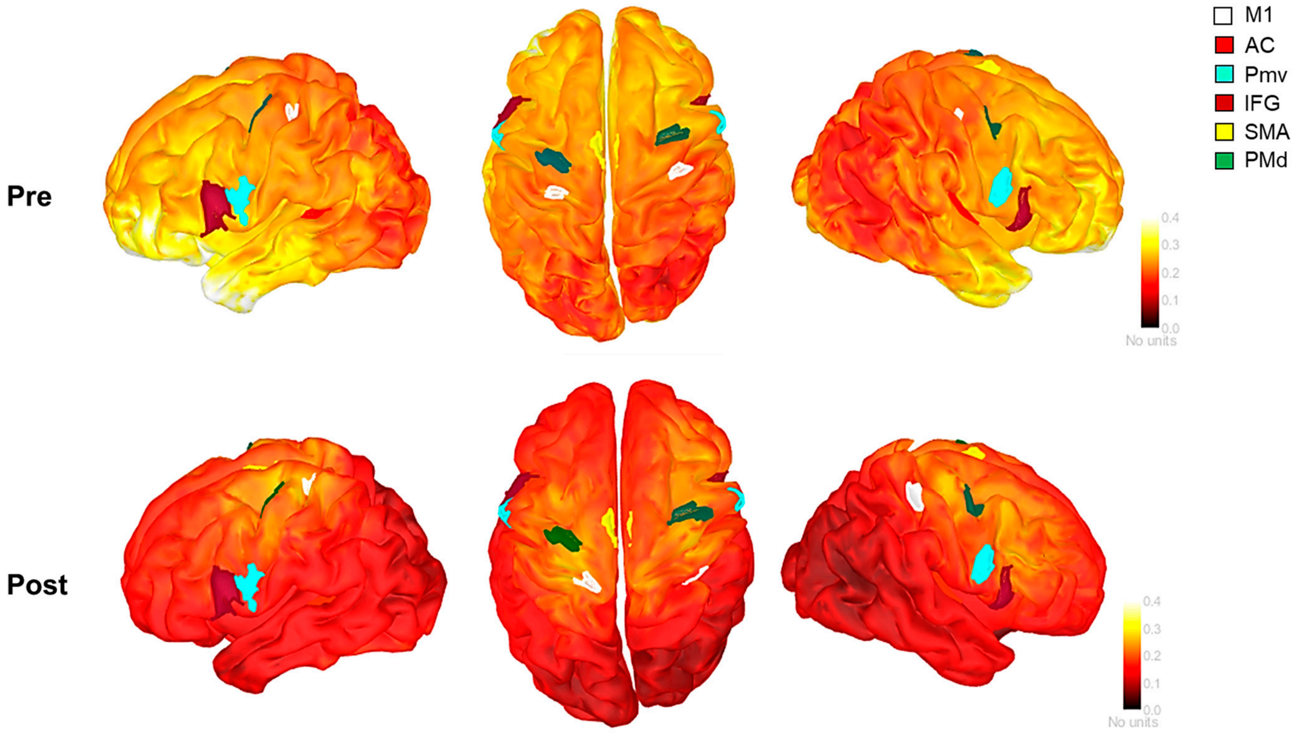

Figure S3. Relative power spectrum density maps for β-band in Participant 1. (M1: primary motor cortex, AC: auditory cortex, IFG: inferior frontal gyrus, SMA: supplementary motor area: PMv: premotor ventral cortex, PMd: premotor dorsal cortex, pre: before music support training, post: after music supported training, post-pre: difference in PSD for the post MST phase and the pre MST phase).

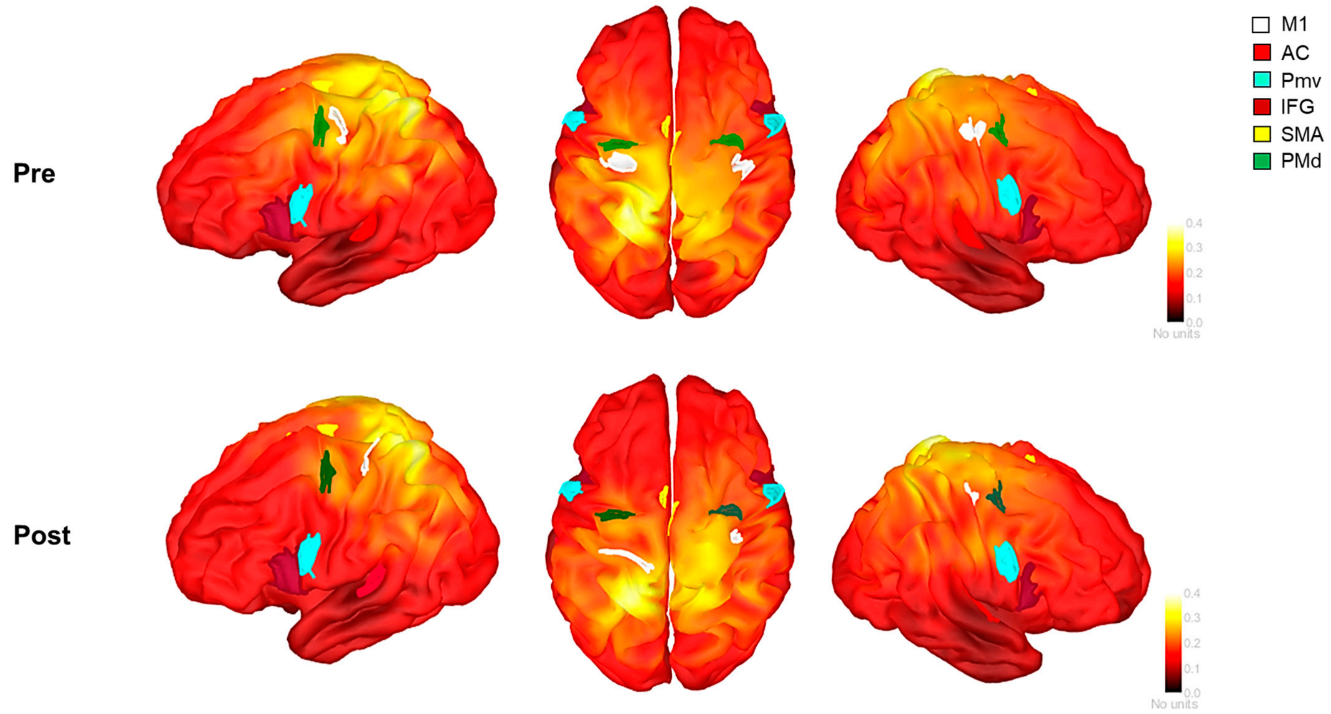

Figure S4. Relative power spectrum density maps for β-band in Participant 2. (M1: primary motor cortex, AC: auditory cortex, IFG: inferior frontal gyrus, SMA: supplementary motor area: PMv: premotor ventral cortex, PMd: premotor dorsal cortex, pre: before music support training, post: after music supported training, post-pre: difference in PSD for the post MST phase and the pre MST phase).

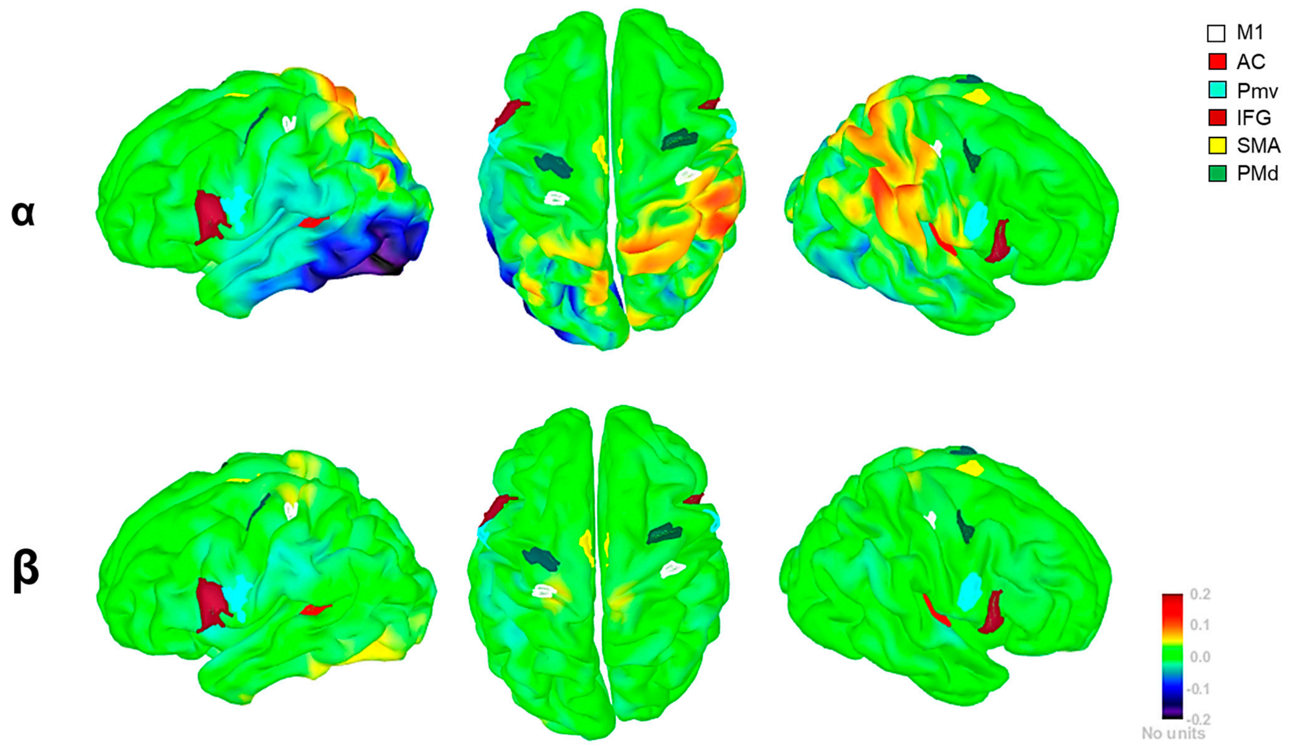

Figure S5. Relative power spectrum density contrast maps for α, β-band in Participant 1. The maps represent a difference in between the post-MST and the pre-MST condition. (M1: primary motor cortex, AC: auditory cortex, IFG: inferior frontal gyrus, SMA: supplementary motor area: PMv: premotor ventral cortex, PMd: premotor dorsal cortex).

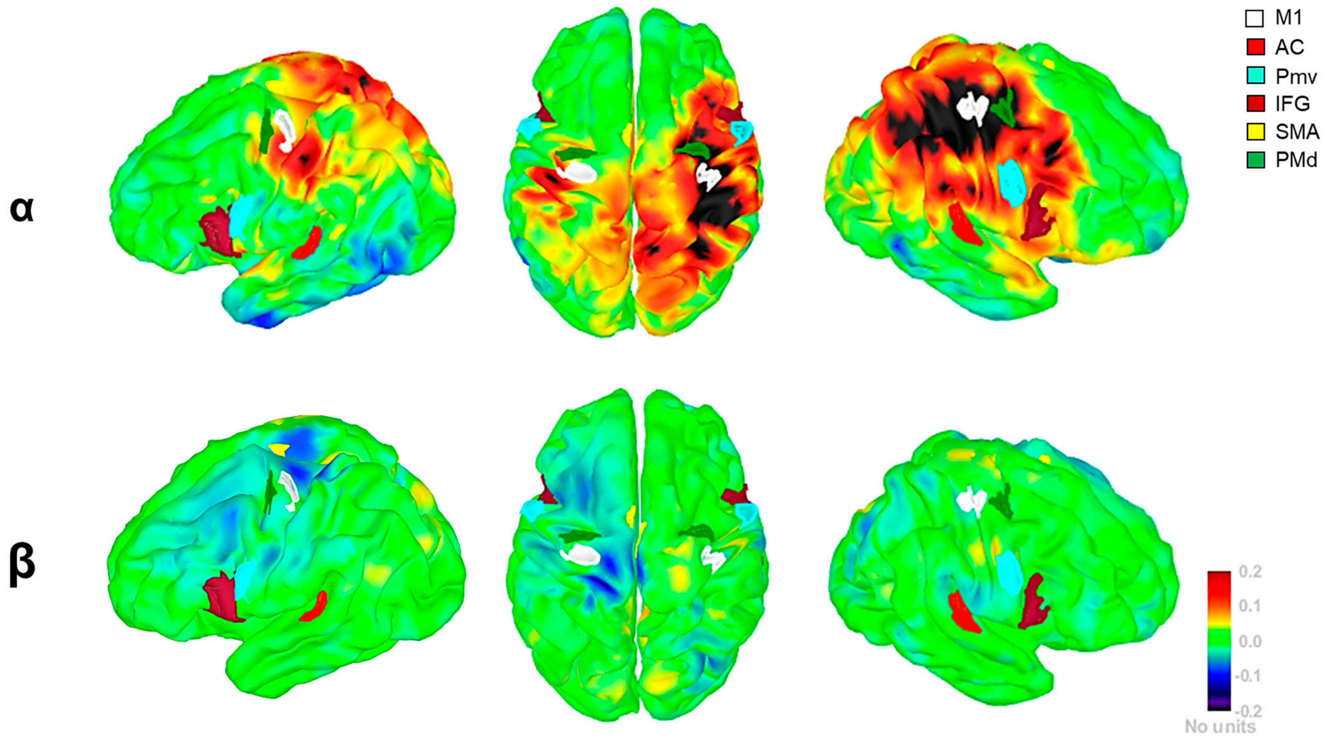

Figure S6. Relative power spectrum density contrast maps for α, β-band in Participant 2. The maps represent a difference in between the post-MST and the pre-MST condition. (M1: primary motor cortex, AC: auditory cortex, IFG: inferior frontal gyrus, SMA: supplementary motor area: PMv: premotor ventral cortex, PMd: premotor dorsal cortex).

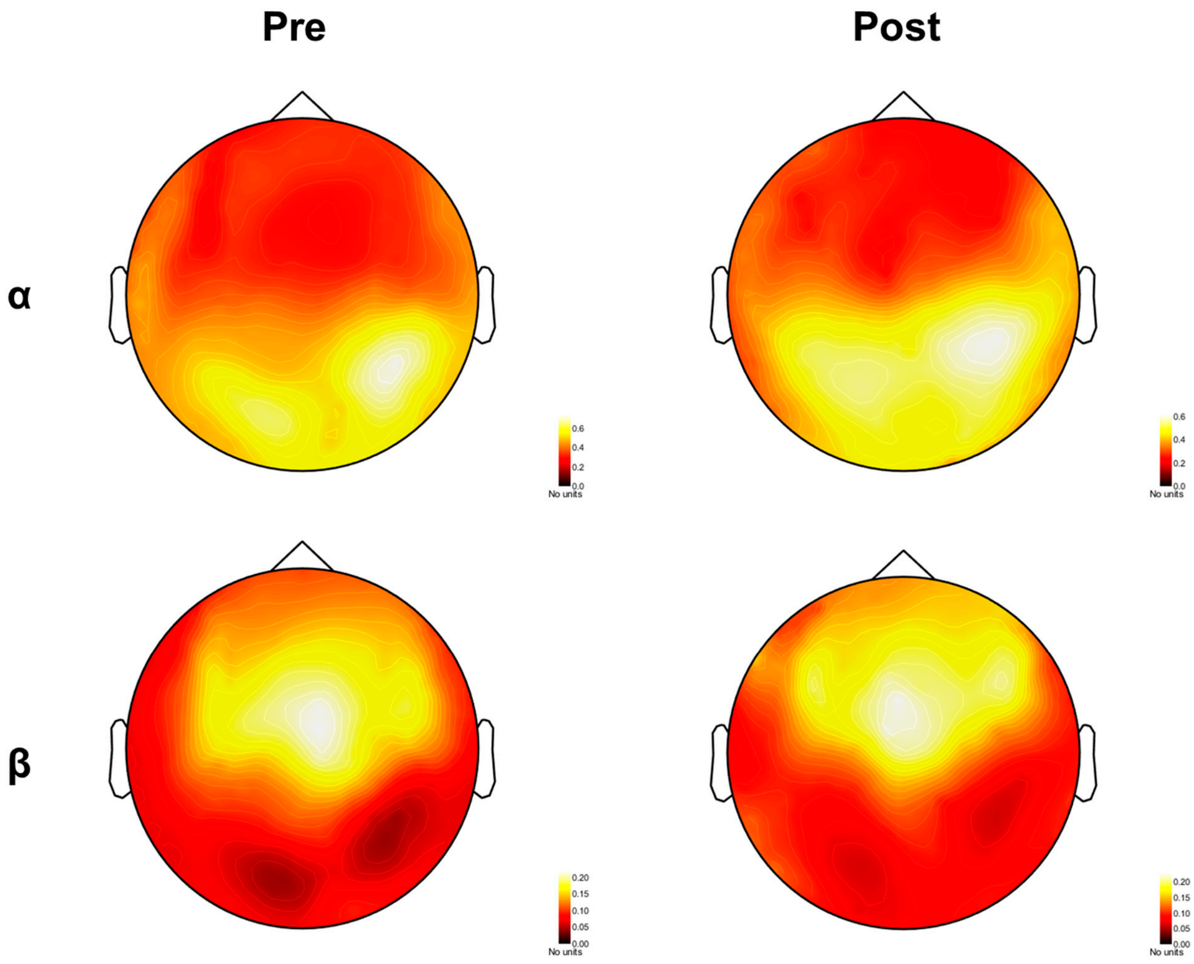

Figure S7. Relative power spectrum density contrast maps (MEG scalp distribution) for α, β-band in Participant 1. (pre: before music support training, post: after music supported training).

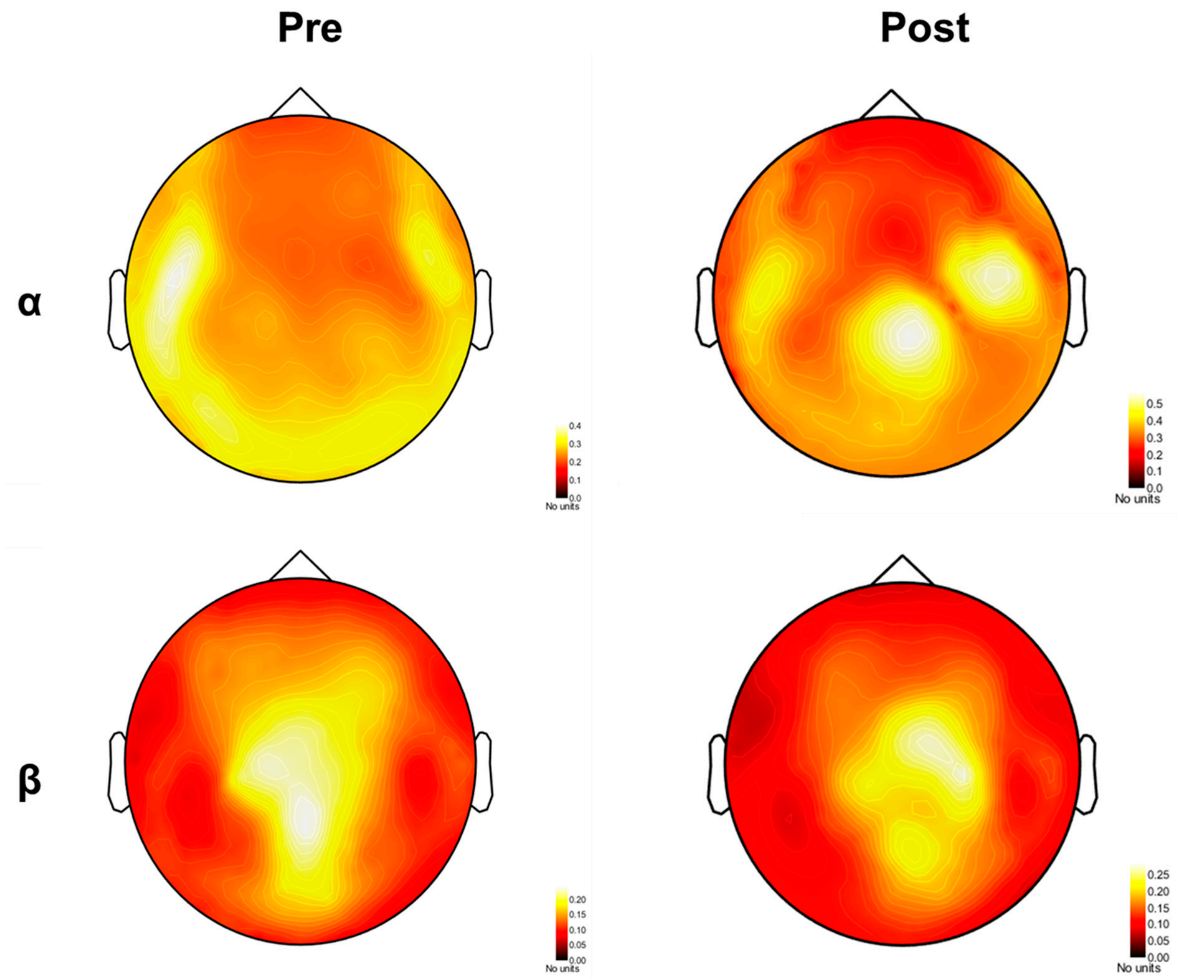

Figure S8. Relative power spectrum density contrast maps (MEG scalp distribution) for α, β-band in Participant 2. (pre: before music support training, post: after music supported training).

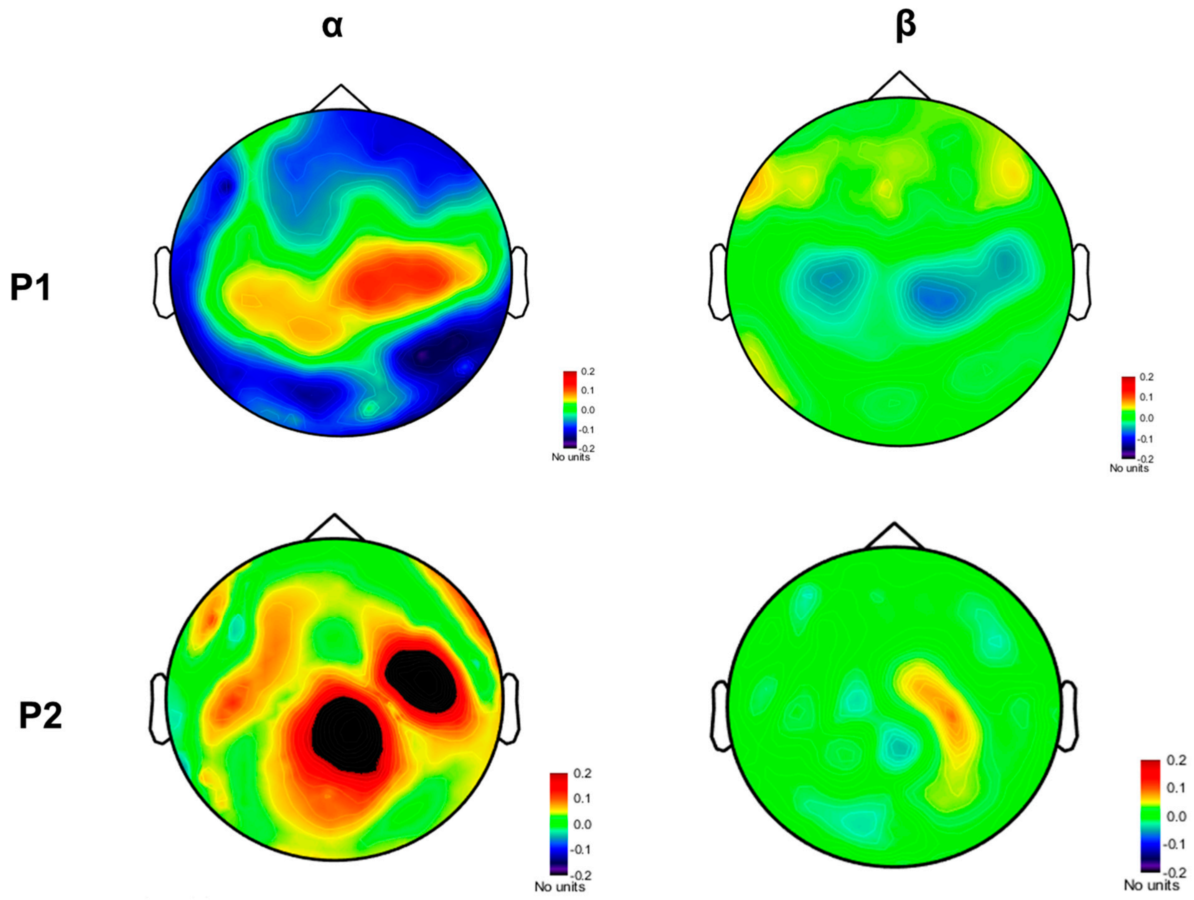

Figure S9. Relative power spectrum density contrast maps (MEG scalp distribution) for α, β-band in Participant 1 and 2. The topographies represent a difference in between the post-MST and the pre-MST condition. (P1: participant 1, P2: participant 2).
